# Prognostic and Predictive Value of Transcription Factors Panel for Digestive System Carcinoma

**DOI:** 10.3389/fonc.2021.670129

**Published:** 2021-10-21

**Authors:** Guoxu Fang, Jianhui Fan, Zongren Ding, Rong Li, Kongying Lin, Jun Fu, Qizhen Huang, Yongyi Zeng, Jingfeng Liu

**Affiliations:** ^1^ Department of Hepatopancreatobiliary Surgery, Mengchao Hepatobiliary Hospital of Fujian Medical University, Fuzhou, China; ^2^ The Big Data Institute of Southeast Hepatobiliary Health Information, Mengchao Hepatobiliary Hospital of Fujian Medical University, Fuzhou, China; ^3^ Department of Hepatology for Pregnancy, Mengchao Hepatobiliary Hospital of Fujian Medical University, Fuzhou, China; ^4^ Department of Pathology, Eastern Hepatobiliary Surgery Hospital of Naval Medical University, Shanghai, China; ^5^ Department of Radiation Oncology, Mengchao Hepatobiliary Hospital of Fujian Medical University, Fuzhou, China; ^6^ Department of Hepatopancreatobiliary Surgery, Fujian Medical University Cancer Hospital, Fujian Cancer Hospital, Fuzhou, China

**Keywords:** digestive system carcinoma, prognosis, transcription factor, overall survival, bioinformatics

## Abstract

**Purpose:**

Digestive system carcinoma is one of the most devastating diseases worldwide. Lack of valid clinicopathological parameters as prognostic factors needs more accurate and effective biomarkers for high-confidence prognosis that guide decision-making for optimal treatment of digestive system carcinoma. The aim of the present study was to establish a novel model to improve prognosis prediction of digestive system carcinoma, with a particular interest in transcription factors (TFs).

**Materials and Methods:**

A TF-related prognosis model of digestive system carcinoma with data from TCGA database successively were processed by univariate and multivariate Cox regression analyses. Then, for evaluating the prognostic prediction value of the model, ROC curve and survival analysis were performed by external data from GEO database. Furthermore, we verified the expression of TFs expression by qPCR in digestive system carcinoma tissue. Finally, we constructed a TF clinical characteristics nomogram to furtherly predict digestive system carcinoma patient survival probability with TCGA database.

**Results:**

By Cox regression analysis, a panel of 17 TFs (NFIC, YBX2, ZBTB47, ZNF367, CREB3L3, HEYL, FOXD1, TIGD1, SNAI1, HSF4, CENPA, ETS2, FOXM1, ETV4, MYBL2, FOXQ1, ZNF589) was identified to present with powerful predictive performance for overall survival of digestive system carcinoma patients based on TCGA database. A nomogram that integrates TFs was established, allowing efficient prediction of survival probabilities and displaying higher clinical utility.

**Conclusion:**

The 17-TF panel is an independent prognostic factor for digestive system carcinoma, and 17 TFs based nomogram might provide implication an effective approach for digestive system carcinoma patient management and treatment.

## Introduction

Digestive system carcinoma, including esophageal, gastric, colon, liver, and pancreatic cancers, is one of the most common malignancies in the world. It is the third leading cause of cancer-related death worldwide and is notorious for its poor prognosis, especially in colon cancer. Despite surgical interventions and other treatments, such as targeted therapy and immunotherapy, 5-year survival rates are not good (1). Prognostic assessment has important clinical significance in guiding the selection of the best treatment plan for patients with digestive system carcinoma. Although clinical outcomes in patients with digestive tract tumors are primarily related to risk factors, including TNM stage, metastasis, and tumor size, these currently used evaluation measures do not provide accurate and individualized prognostic information to facilitate effective treatment selection. Therefore, it is important to study effective prognostic predictors that influence overall survival (OS), and we should pay more attention to digestive system carcinoma and it is urgent to develop new methods to treat gastrointestinal digestive system carcinoma.

Transcription factor (TF) is an important component of transcription regulation. TF is a kind of DNA binding protein, which can bind to specific DNA sequence and then affect the genetic information from DNA transcription to RNA. In recent years, with the development of chip and high-throughput sequencing technology and the rapid development of bioinformatics, a large number of transcription factor databases have been generated, which are very important for gene transcriptional regulation and TF-related molecular biology research. With the in-depth study of the gene expression mechanism, studies have confirmed that the imbalance of TF is an important pathological basis for the occurrence of cancer (2). In this paper, TCGA databases (training cohorts) were used to search for transcription factors related to tumor prognosis and establish a prediction model. Then, GEO databases (validation cohorts) were used for validation. Our results suggest that transcription factors are important factors affecting the prognosis of cancer patients and may be potential targets for the treatment of digestive system cancer.

## Materials and Methods

### Data Download and Preprocessing

The TCGA-esophagus cancer dataset consisted of the RNA-seq data of 160 esophageal cancer tissue and 11 adjacent normal samples, and related clinical characteristics were downloaded from the TCGA database. The TCGA-gastric cancer dataset consisted of the RNA-seq data of 381gastric cancer tissue, and 32 adjacent normal samples and related clinical characteristics were downloaded from the TCGA database. The TCGA-colon cancer dataset consisted of the RNA-seq data of 482 colon cancer tissue, and 42 adjacent normal samples and related clinical characteristics were downloaded from the TCGA database. The TCGA-liver cancer dataset consisted of the RNA-seq data of 374 liver cancer tissue, and 50 adjacent normal samples and related clinical characteristics were downloaded from the TCGA database. The TCGA-pancreatic cancer dataset consisted of the RNA-seq data of 178 pancreatic cancer tissue, and four adjacent normal samples and related clinical characteristics were downloaded from the TCGA database. GSE53624 (esophagus cancer dataset), GSE84433 (gastric cancer dataset), GSE40967(colon cancer dataset), GSE10143 (liver cancer dataset), and GSE57495 (pancreatic cancer dataset), which contained the gene expression data with clinical characteristics, were downloaded from the GEO database (http://ncbi.nlm.nih.gov/geo/), respectively. The raw data were preprocessed with the following criteria: (1) the genes were excluded if the FPKM value (Fragments per Kilobase Million) was zero in more than half of the samples; (2) genes with missing expression values in more than 30% of samples were removed; (3) the invariant genes (i.e., same expression value across all samples) and low-variation genes were filtered; (4) samples without related clinical data or overall survival (OS) <30 days were removed. The TCGA datasets were enrolled as a training group, and the GEO datasets were regarded as the external validation cohorts. As the data were open-access, therefore, the ethical approval by an ethics committee was not required.

### Differentially Expressed TFs Identification

DETFs identification: The R package of “DESeq2” was used to calculate the DEGs of the cancer and normal tissue [FDR (false discovery rate) <0.05 and |log2FC (fold change)| >1].

### Statistical Analysis

R software (version 4.0.2) and SPSS software (version 22.0) were used to complete all the statistic work. OS was calculated by the KM method, and the differences between the groups were compared by using the log-rank test. Cox proportional hazard model was used to analyze the significant transcription factors affecting OS. *P* < 0.05 was considered statistically significant.

### Risk Model Construction and Validation

The expression data of the transcription factors associated with prognosis in the training cohorts (TGCA datasets) were used in constructing a risk score model. External validation cohorts (GEO datasets) were then used to verify the reliability of the risk score model. The risk score of each sample was calculated based on formula. Then, the samples were divided into high-risk and low-risk groups by the risk score median. The risk score distribution was plotted by the R package of “time ROC (receiver operating characteristic)”. A log-rank test was used in comparing the survival difference between the two groups. The overall survival (OS) of each group was performed using the Kaplan–Meier (KM) survival curve.

### Gene Set Enrichment Analysis

GSEA enrichment in the TCGA datasets were conducted for the analysis of the significantly enriched pathways in the transcription factors associated with prognosis. c2.cp.kegg.v7.2 symbols were selected for our analysis, which included the KEGG pathways database.

### Sample Collection

Cancer tissues and adjacent tissues were collected from 150 patients. The study was approved by the Ethics Committee of Eastern Hepatobiliary Surgery Hospital. Informed consent was obtained from all patients before surgery for using their data in the research.

### Quantitative Reverse Transcription Polymerase Chain Reaction

Cell total RNA was extracted using Trizol reagent (Invitrogen, USA) following the manufacturer’s instructions. The quantity and quality of extracted RNA were assessed by the spectrophotometric (Dojindo Laboratories, Kumamoto, Japan) determination of absorbance ratio (A260/A280). Then, the prepared RNA was reversely transcribed into cDNA using reverse transcriptase (Invitrogen, USA) and random primers. One microliter of synthesized cDNA was used in each qPCR reaction. SYBR Green-based qRT-PCR was subsequently executed on ABI PRISM 7300HT Sequence Detection System (Applied Biosystems, USA). β-Actin was used as a control for normalization. Primers used in RT-PCR were as **Table 1**.

**Table 1:** Primer sequences of 17 TFs associated with digestive system tumor prognosis.

gene	Forward Sequence	Reverse Sequence
CENPA	GGCGGAGACAAGGTTGGCTAAA	GGCTTGCCAATTGAAGTCCACAC
ETS2	ACTCCGCCAACTGTGAATTGCC	CCACTGGCATACCTGTTGCTCA
MYBL2	CACCAGAAACGAGCCTGCCTTA	CTCAGGTCACACCAAGCATCAG
FOXM1	TCTGCCAATGGCAAGGTCTCCT	CTGGATTCGGTCGTTTCTGCTG
ETV4	AGGAACAGACGGACTTCGCCTA	CTGGGAATGGTCGCAGAGGTTT
FOXQ1	CCTACTCGTACATCGCGCTCAT	TCGTTGAGCGAAAGGTTGTGGC
ZNF589	TGGCTGTGCTTTTCACTGAGGC	AAGGGCAGGTATGGACTTCTGG
NFIC	TGGCGGCGATTACTACACTTCG	GGCTGTTGAATGGTGACTTGTCC
YBX2	GATGTCGTGGAAGGAGAGAAGG	GATGAATCGGCGGGACTTACGT
ZBTB47	CAATGGTGCGGCAAGGACTTCA	CTGGTGAAGCTCTTGCCACAGA
ZNF367	GGACAGCTCAAAACACATCAGCG	TTCGGACAGTGGCGGTTTGCAT
CREB3L3	GAAGCCTCTGTGACCATAGACC	GGAGGTCTTTCACGGTGAGATTG
HEYL	TGGAGAAAGCCGAGGTCTTGCA	ACCTGATGACCTCAGTGAGGCA
FOXD1	GATCTGTGAGTTCATCAGCGGC	TGACGAAGCAGTCGTTGAGCGA
TIGD1	TCATTGACGAAGGTGGCTACACT	GCTTTGAAGCCAGGCACTGACT
SNAI1	TGCCCTCAAGATGCACATCCGA	GGGACAGGAGAAGGGCTTCTC
HSF4	GGACCAGTTTCCTCGTAAGCGA	CTCACCACCTTCCGAAAACCGT

### Bioinformatics Analysis

GO and KEGG functional enrichment analyses were conducted based on the target genes.

## Results

### Identified Differentially Expressed TFs Between Digestive System Carcinoma Tissues and Adjacent Normal Tissues

**Figure 1:**
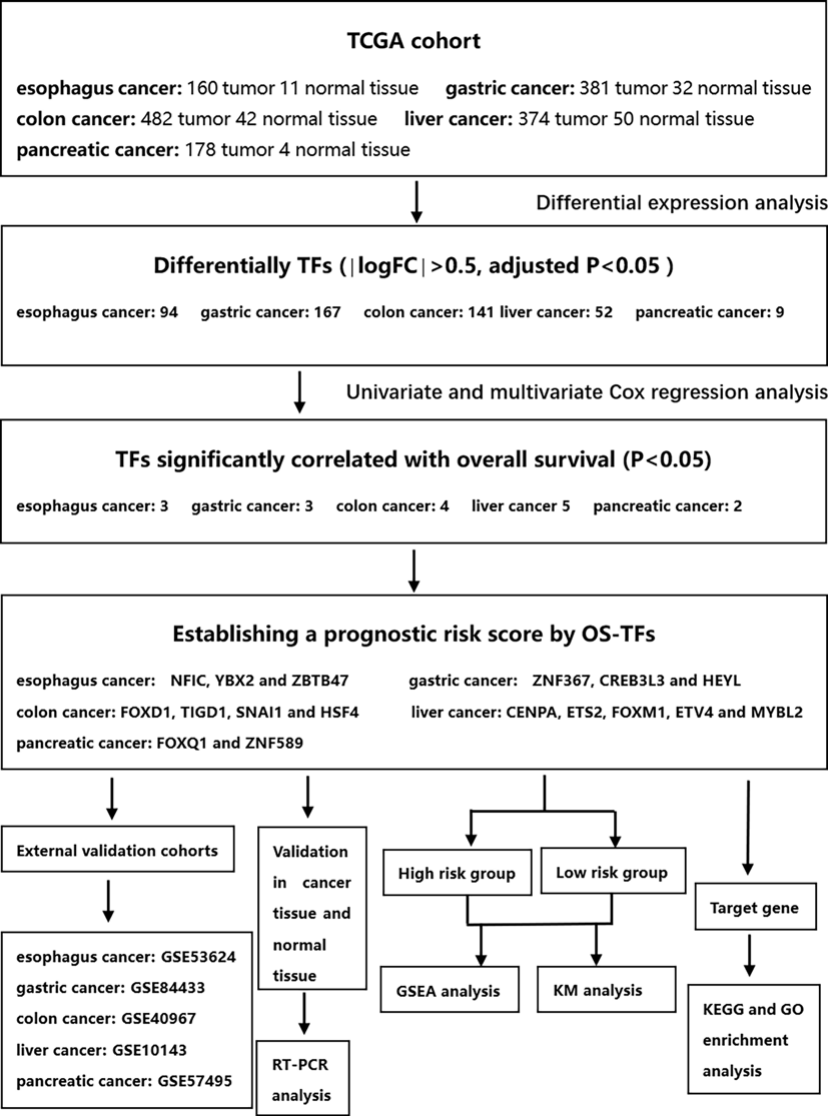
Whole procedure for analyzing TFs in digestive system carcinoma.

**Figure 2:**
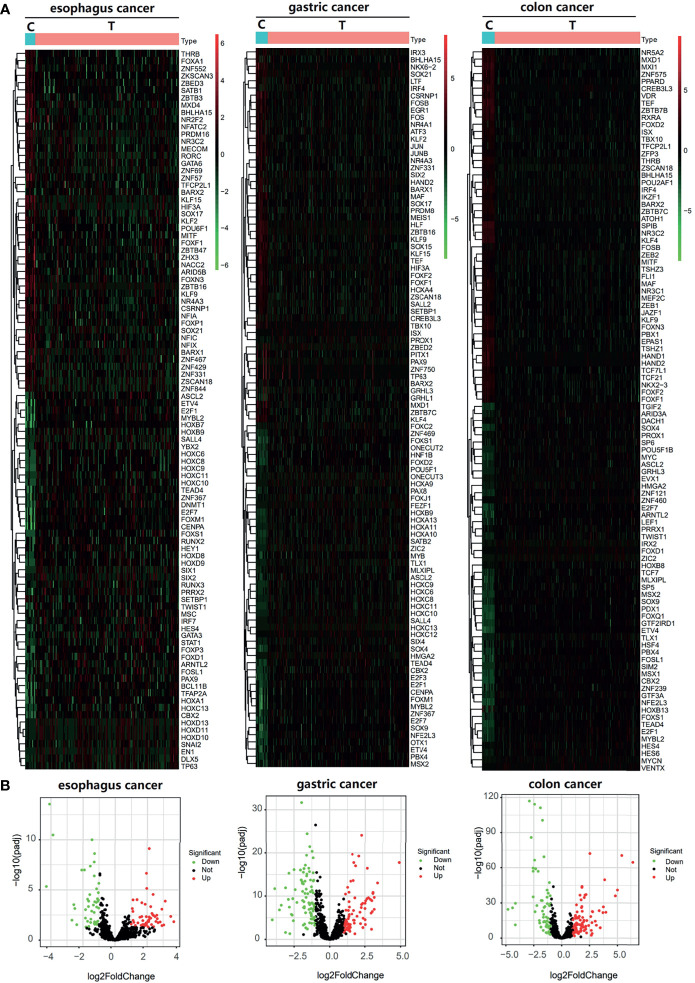
Differential TFs between cancer tissue and paracancer tissue. **(A, B)** Heatmap **(A)** and Volcano plot **(B)** of the differential TFs in the cancer tissue and paracancer tissue of TCGA database.

This study was conducted according to the flow chart shown in **Figure 1**. We found 99, 167, 146, 120, and 9 differential TFs in normal paracancer samples and tumor samples of esophageal cancer, gastric cancer, colon cancer, liver cancer, and pancreatic cancer, respectively, by analyzing the TCGA database. After intersection with the GEO database, we found 94, 167, 141, 52, and 9 common differential TFs in the normal paracancer samples and tumor samples of esophageal cancer, gastric cancer, colon cancer, liver cancer, and pancreatic cancer, respectively (**Figures 2A, B** and **Supporting Figures S1A, B**).

### Identified OS-Related TFs in DETFs

**Figure 3:**
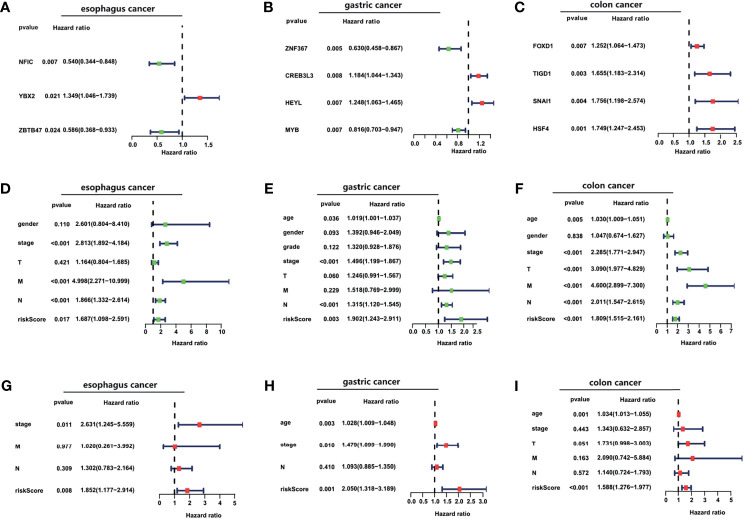
The construction of TFs signature and the evaluation of its independent prognostic value. **(A–C)** Forest plot of the univariate Cox regression analysis with TFs in esophageal cancer, gastric cancer, and colon cancer. **(D–I)** Forest plots of the univariate **(D–F)** and multivariate **(G–I)** Cox regression analysis with clinical features and risk score in TCGA cancer cohorts.

In esophageal cancer, OS-related TFs NFIC, YBX2, and ZBTB47 were obtained by univariate and multivariate Cox regression analyses (**Figure 3A**). In gastric cancer, OS-related TFs ZNF367, CREB3L3, HEYL, and MYB were obtained by univariate Cox regression analysis. ZNF367, CREB3L3, and HEYL were obtained by multivariate Cox regression analysis (**Figure 3B**). In colon cancer, OS-related TFs FOXD1, TIGD1, SNAI1, and HSF4 were obtained by univariate and multivariate Cox regression analyses (**Figure 3C**). In liver cancer, OS-related TFs CENPA, HMGA1, ETS2, FOXO1 KLF9, AR, FOXM1, ETV4, MYBL2, ZIC2 were obtained by univariate Cox regression analysis. CENPA, ETS2, FOXM1, ETV4, MYBL2 were obtained by multivariate Cox regression analysis (**Supporting Figure S2A**). In pancreatic cancer, OS-related TFs SPDEF, FOXQ1, ZNF589 were obtained by univariate Cox regression analysis. FOXQ1 and ZNF589 were obtained by multivariate Cox regression analysis (**Supporting Figure S2B**).

A prognostic risk score for each patient was calculated based on the mRNA expression levels of the OS-related TFs and the coefficients from univariate and multivariate Cox regression analyses. We performed univariate and multivariate Cox regression analyses to evaluate the prognostic value of the risk score. The forest plot was utilized to show the clinical features such as age, gender, and the tumor TNM stage in the nomogram, as shown in **Figures 3D–I** and **Supporting Figures S2C–E**. As shown in **Figures 3D–F** and **Supporting Figures S2C, D**, the hazard ratio of risk score, which was performed by univariate Cox regression analysis, is around 1.687, 1.902, 1.809, 1.977, 1.921. As shown in **Figures 3G–I**, the risk score, performed by multivariate Cox regression analysis, is around 1.852, 2.050, 1.588. Our data analysis shows that risk score is an independent risk factor for the prognosis of esophageal cancer, gastric cancer, and colon cancer.

### Construct TF-Related Prognostic Model of Digestive System Carcinoma

**Figure 4:**
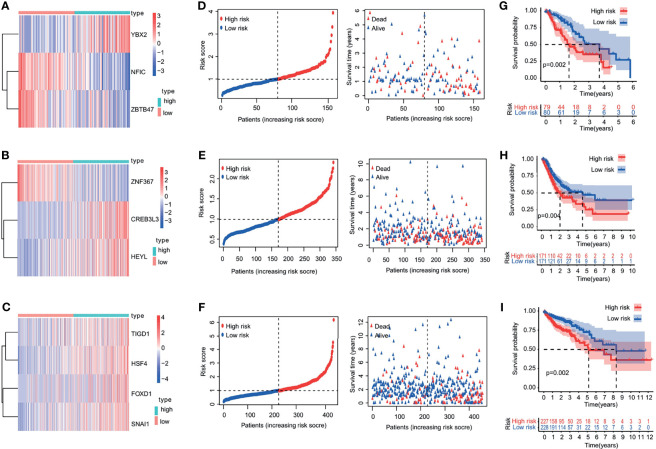
Heatmap, characteristics of the risk score, and Kaplan-Meier analysis of the OS-related TFs in training cohorts (TCGA database). **(A–C)** Heatmap of the gene-expression profiles of OS-related TFs in training cohorts: **(A)** esophageal cancer, **(B)** gastric cancer, **(C)** colon cancer. **(D–F)** The distributions of the risk score, survival time, and status of patients in training cohorts: **(D)** esophageal cancer, **(E)** gastric cancer, **(F)** colon cancer. **(G–I)** Kaplan-Meier curves of OS-related TFs in training cohorts: **(G)** esophageal cancer, **(H)** gastric cancer, **(I)** colon cancer.

The patients were divided into a low-risk group and a high-risk group, according to the median value of the risk scores in the TCGA training cohorts. The gene-expression profiles of the prognostic risk genes between the high-risk group and low-risk group are displayed in the heatmap in **Figures 4A–C** and **Supporting Figures S3A, B**. The distribution of the risk scores and the correlation between the risk scores and survival data are illustrated in scatterplots (**Figures 4D–F** and **Supporting Figures S3C, D**). K-M survival analysis revealed a significantly higher survival probability in the low-risk group (p < 0.05) (**Figures 4G–I** and **Supporting Figures S3E, F**). Patients with high-risk scores were associated with significantly worse OS, thereby suggesting that the high-risk score was an adverse prognostic factor.

### Validate TF-Related Prognostic Model of Digestive System Carcinoma

**Figure 5:**
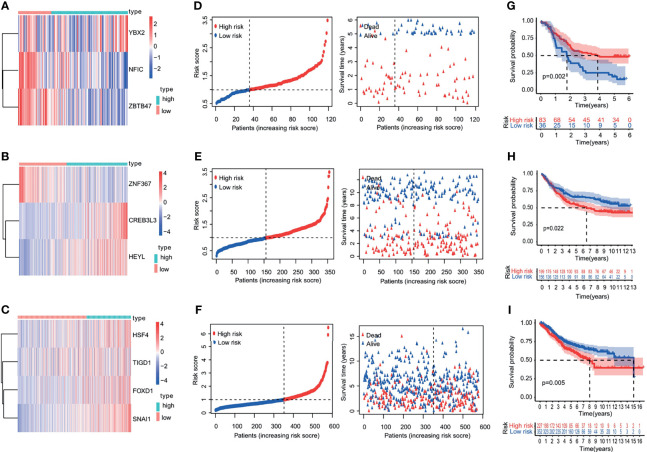
Heatmap, characteristics of the risk score, and Kaplan-Meier analysis of the OS-related TFs in validation cohorts (GEO database). **(A–C)** Heatmap of the gene-expression profiles of OS-related TFs in validation cohorts: **(A)** esophageal cancer, **(B)** gastric cancer, **(C)** colon cancer. **(D–F)** The distributions of the risk score, survival time, and status of patients in validation cohorts: **(D)** esophageal cancer, **(E)** gastric cancer, **(F)** colon cancer. **(G–I)** Kaplan-Meier curves of OS-related TFs in validation cohorts: **(G)** esophageal cancer, **(H)** gastric cancer, **(I)** colon cancer.

The robustness of the risk model was further assessed with external datasets using the GEO datasets of GSE53624 (esophagus cancer data), GSE84433 (gastric cancer data), GSE40967 (colon cancer date), GSE10143 (liver cancer date), and GSE57495 (pancreatic cancer date), respectively. The gene-expression profiles of the validation cohorts are visualized in **Figures 5A–C** and **Supporting Figures S4A, B**. The distribution of the risk scores and the correlation between the risk scores and survival data of validation cohorts are illustrated in **Figures 5D–F** and **Supporting Figures S4C, D**. The K-M survival curves revealed a higher survival probability of the low-risk group (p <0.05) in validation cohorts (**Figures 5G–I** and **Supporting Figures S4E, F**). Similar results that high-risk score was related to worse OS were obtained in validation cohorts. The model had a relatively high distinguishing ability of prognosis and could identify the high-risk group patients with worse survival results in validation cohorts.

### Exploration of Signaling Pathways

**Figure 6:**
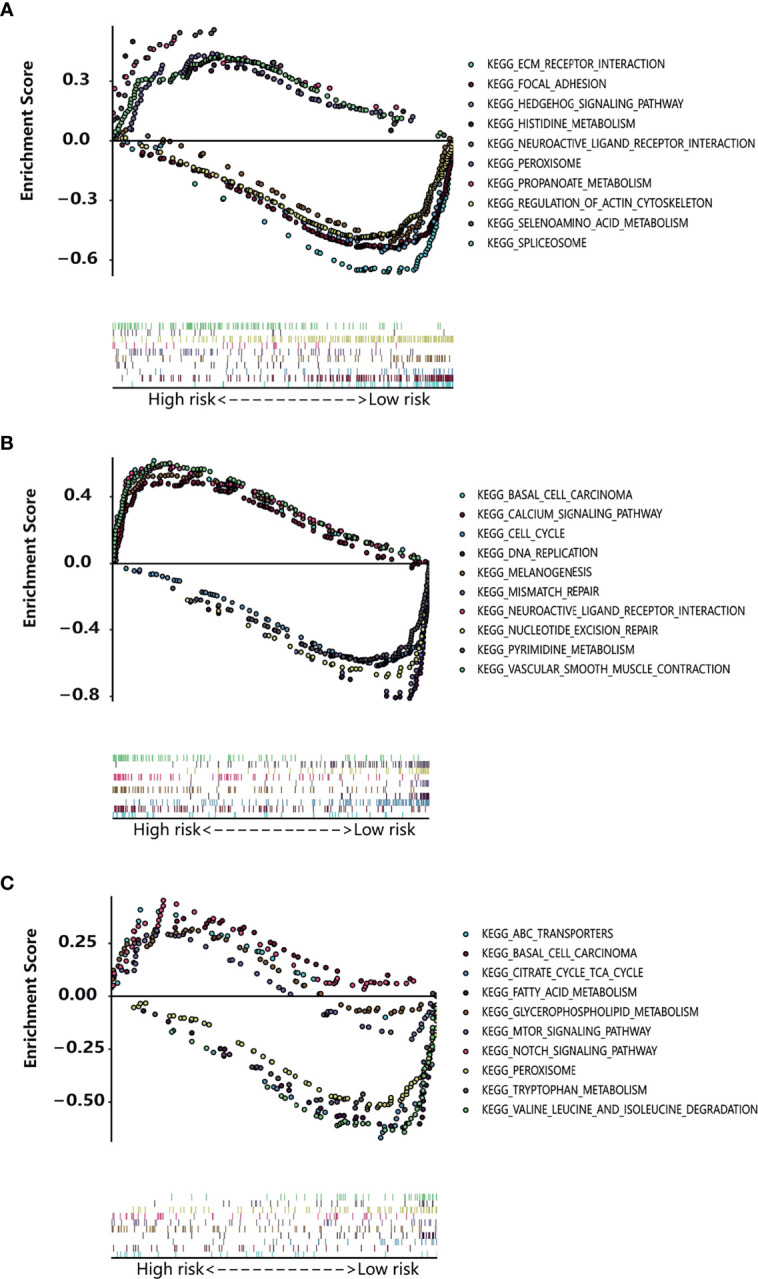
Enrichment plot of the OS-related TFs between the high-risk and low-risk groups using GSEA. **(A–C)** The enriched gene sets in KEGG collection in esophagus cancer sample **(A)**, gastric cancer sample **(B)**, and colon cancer sample **(C)** with high risk score and low risk score.

Gene set enrichment analysis (GSEA) has an advantage in exploring the involved signaling pathways. GSEA revealed that the genes in the high-risk group of TCGA cohorts were significantly enriched in selenoamino acid metabolism, peroxisome, histidine metabolism (esophageal cancer); neuroactive ligand receptor interaction, vascular smooth muscle contraction, melanogenesis (gastric cancer); glycosaminoglycan biosynthesis chondroitin sulfate, notch signaling pathway, ABC transporters (colon cancer). In contrast, the low-risk group genes were significantly enriched in pathways such as neuroactive ligand receptor interaction, regulation of actin cytoskeleton, focal adhesion (esophageal cancer); cell cycle, nucleotide excision repair, pyrimidine metabolism (gastric cancer); valine leucine and isoleucine degradation, peroxisome, fatty acid metabolism (colon cancer) (**Figures 6A–C**).The results of OS-related TFs enrichment analysis for liver cancer and pancreatic cancer are shown in **Supporting Figures S5A, B** and **Table 2**.

**Table 2:** The result of KEGG enrichment analysis of high-risk group and low-risk group in liver cancer and pancreatic cancer.

liver cancer	high-risk group	low-risk group
	KEGG_CELL_CYCLE	KEGG_COMPLEMENT_AND_COAGULATION_CASCADES
	KEGG_DNA_REPLICATION	KEGG_PPAR_SIGNALING_PATHWAY
	KEGG_MISMATCH_REPAIR	KEGG_FATTY_ACID_METABOLISM
	KEGG_SPLICEOSOME	KEGG_PEROXISOME
	KEGG_OOCYTE_MEIOSIS	KEGG_RETINOL_METABOLISM
**pancreatic cancer**	**high-risk group**	**low-risk group**
	KEGG_SELENOAMINO_ACID_METABOLISM	KEGG_REGULATION_OF_ACTIN_CYTOSKELETON
	KEGG_PEROXISOME	KEGG_NEUROACTIVE_LIGAND_RECEPTOR_INTERACTION
	KEGG_HISTIDINE_METABOLISM	KEGG_ECM_RECEPTOR_INTERACTION
	KEGG_SPLICEOSOME	KEGG_FOCAL_ADHESION
	KEGG_PROPANOATE_METABOLISM	KEGG_DILATED_CARDIOMYOPATHY

### Nomogram Construction

**Figure 7:**
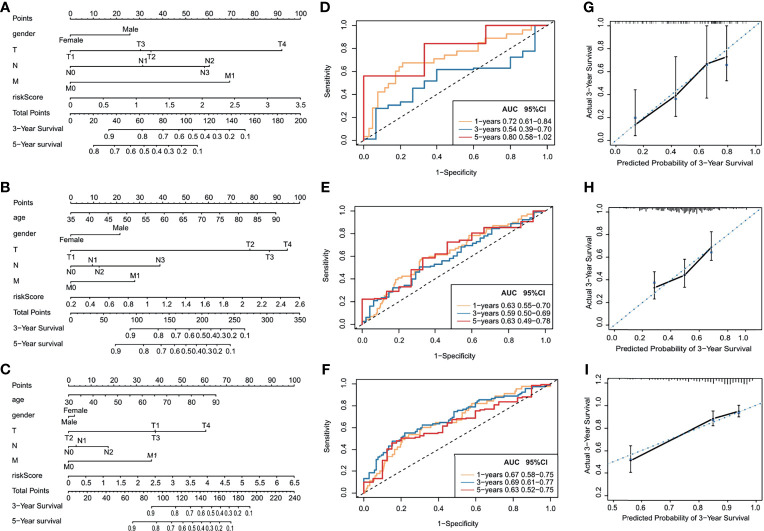
Prognostic capacity evaluation and nomogram analysis of panel of OS-TFs of digestive system carcinoma patients. **(A–C)** Nomogram predicting the OS in digestive system carcinoma patients containing the risk score: **(A)** esophageal cancer, **(B)** gastric cancer, **(C)** colon cancer. **(D–F)** ROC curves and AUC for 1-, 3-, and 5-year survival of the nomogram: **(D)** esophageal cancer, **(E)** gastric cancer, **(F)** colon cancer. **(G–I)** Calibration curve of 3-year survival in the nomogram and ideal model: **(G)** esophageal cancer, **(H)** gastric cancer, **(I)** colon cancer.

TCGA training cohort was used to screen the prognostic risk factors. The risk score, existing the inducement to digestive system carcinoma, age, gender, T stage, N stage, M stage were found to be risk factors for OS after the univariable and Cox regression analyses. Then, a nomogram model with a C-index value of 0.763 (95% CI=0.693–0.831) in esophageal cancer, 0.658 (95% CI=0.607–0.708) in gastric cancer, 0.785 (95% CI=0.729–0.843) in colon cancer, 0.738 (95% CI=0.683–0.793) in liver cancer, 0.644 (95% CI=0.545–0.742) in pancreatic cancer, containing the above clinical features and risk score was constructed, as shown in **Figures 7A–C** and **Supporting Figures S6A, B**.

Furthermore, the score for each patient was calculated according to the nomogram, and the prediction accuracy of the nomogram was assessed using the ROC curve. The AUCs of the nomogram model are shown in **Figures 7D–F** and **Supporting Figures S6C, D**. **Figures 7G–I** and **Supporting Figures S6E, F** show the calibration curve between the nomogram and the ideal model. Results show that the model was consistent with the ideal model, indicating that the accuracy of our model was relatively high.

### Validated OS-Related TFs Between Digestive System Carcinoma Tissues and Adjacent Normal Tissues

**Figure 8:**
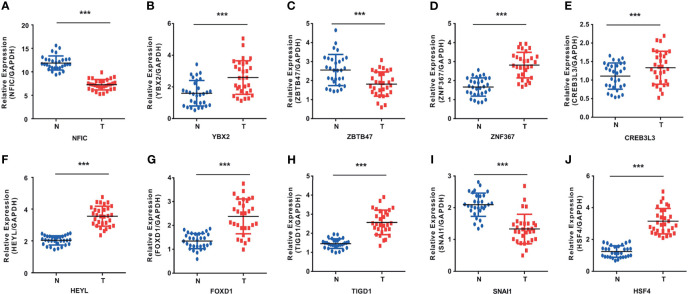
The results of qRT-PCR in OS-related TFs. **(A–C)** YBX2 was highly expressed and NFIC and ZBTB47 were lowly expressed in esophageal cancer tissues. **(D–F)** ZNF367, CREB3L3, and HEYL were highly expressed in gastric cancer tissues. **(G–J)** FOXD1, TIGD1, and HSF4 were highly expressed and SNAI1 was lowly expressed in colon cancer tissues. ***p < 0.001.

To validate whether NFIC, ZBTB47, SNAI1 are lowly expressed and YBX2, ZNF367, CREB3L3, HEYL, FOXD1, TIGD1, HSF4 are highly expressed in cancer tissues, we experimentally validated OS-related TFs expression in the tissues of 30 esophageal cancer patients, 30 gastric cancer patients, and 30 colon cancer patients. The results of qRT-PCR suggested that NFIC, ZBTB47, SNAI1 are lowly expressed and YBX2, ZNF367, CREB3L3, HEYL, FOXD1, TIGD1, HSF4 are highly expressed in cancer tissues (**Figures 8A–J**). The expressions of liver cancer and pancreatic cancer OS-related TFs in cancer tissues and adjacent normal tissues are shown in **Supporting Figures S7A–G**.

### Functional Enrichment Analysis of the Target Genes of OS-Related TFs

**Figure 9:**
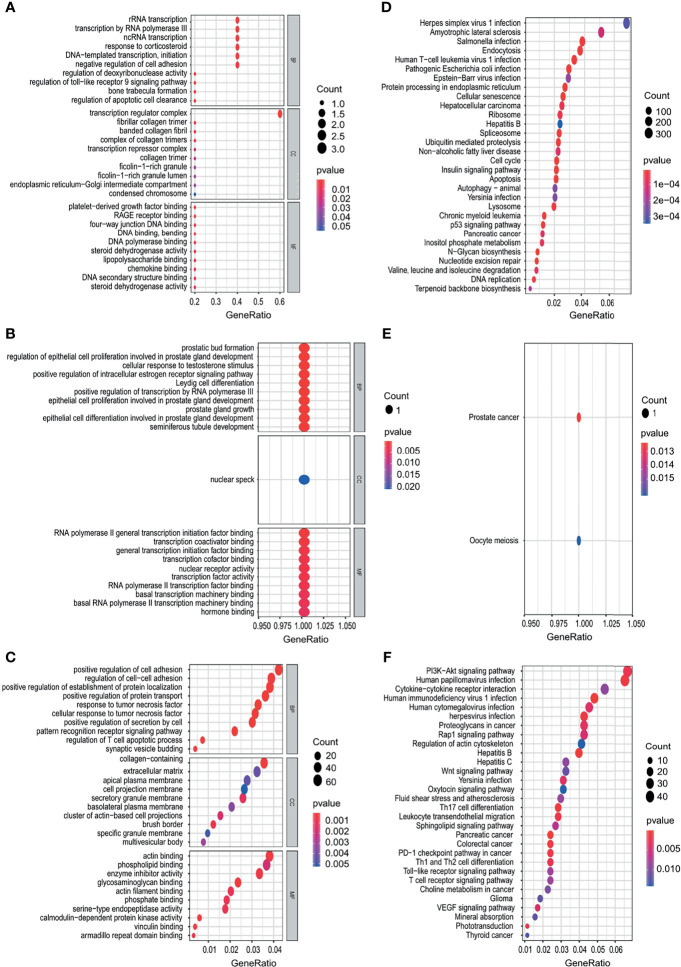
Functional enrichment analysis of the target genes of OS-related TFs. **(A–C)** GO enrichment analysis results, showing only the first 20 terms in esophagus cancer **(A)**, gastric cancer **(B)**, and colon cancer **(C)**. **(D–F)** KEGG pathway enrichment analysis results, showing only the first 20 pathways in esophagus cancer **(D)**, gastric cancer, **(E)**, and colon cancer **(F)**. Gene Ratio refers to the ratio of the number of genes enriched in the term/pathway to the total number of genes in the term/pathway.

The target genes of OS-related TFs were predicted using the CHEA, Encode, Jaspar Motifmap, Transfac, Trusting transcription factor databases. The results are shown in **Supplementary Table 1**. The GO and KEGG enrichment analysis of the target genes was performed. The results showed that the target genes of OS-related TFs were mainly enriched in rRNA transcription, transcription by RNA polymerase III, ncRNA transcription (esophageal cancer); prostatic bud formation, regulation of epithelial cell proliferation involved in prostate gland development, cellular response to testosterone stimulus (gastric cancer); positive regulation of cell adhesion, regulation of cell−cell adhesion, positive regulation of establishment of protein localization (colon cancer) (**Figures 9A–C**) and pathways related to the herpes simplex virus 1 infection, amyotrophic lateral sclerosis, salmonella infection (esophageal cancer); oocyte meiosis (gastric cancer); PI3K−Akt signaling pathway, human papillomavirus infection, cytokine−cytokine receptor interaction (colon cancer) (**Figures 9D–F**). The results of target genes of OS-related TFs enrichment analysis for liver cancer and pancreatic cancer are shown in **Supporting Figures S8A–D**.

## Discussion

The prognosis of digestive system carcinoma patients usually is not good, because the majority of digestive system carcinoma patients have been in an advanced stage when they are diagnosed. Even if advanced digestive system carcinoma patients receive chemotherapy or molecular-targeted drug therapy, the effect is usually poor. With the development of tumor molecular biology, prognostic markers that reflect tumor progression at the molecular level may help to achieve more accurate individualized survival prediction and treatment (3). Recently, molecular prognostic markers have drawn more and more attention of researchers in the survival prediction of digestive system carcinoma (4). We can use them to dynamically detect disease progression or changes in the prognosis of tumor patients. Moreover, compared with a single one marker, a panel of molecular markers will significantly increase the accuracy in reflecting the prognosis of digestive system carcinoma (5).

In the human genome, there are more than 35,000 genes expressed differently in different tissues and at different times. TF plays an important role in the regulation of gene expression and is also a target of signal transduction pathway. More and more researchers begin to study TFs. They think that TFs may be the target of diagnosis and treatment of human diseases. The combination of bioinformatics and molecular biology, that is, the combination of “dry” and “wet” experiments, has become the necessary means to solve various biological problems and has made good progress. TF-related database plays an important role in life science research, which is also the fundamental reason for the frequent development and use of TF-related database in recent years (6).

The results of our study indicate that NFIC, YBX2, ZBTB47 were closely related to the prognosis of esophageal cancer. H. Wang illustrated that the importance of miR-550a-3/NFIC in the regulation of esophageal squamous cell cancer cells growth and metastasis, which could contribute to developing novel targets for early diagnosis or neoteric therapeutic target for esophageal squamous cell cancer (7). G. Xu found that LBX2-AS1 upregulated by NFIC promoted gastric cancer progression *via* targeting miR-491-5p/ZNF703 (8). F. Chen’s research indicated that LINC00958 regulated miR-627-5p/YBX2 axis to facilitate cell proliferation and migration in oral squamous cell carcinoma (9). X. Niu found that LncRNA HOXA11-AS promotes oral squamous cell carcinoma progression by sponging miR-98-5p to upregulate YBX2 expression (10). M. Tan found that the expression of ZBTB47 was different in familial pancreatic cancer predisposed individuals, sporadic pancreatic cancer patients, and normal donor pancreatic tissue (11).

The results of our study indicate that ZNF367, CREB3L3, HEYL were closely related to the prognosis of gastric cancer. Several members of the zinc finger protein family have been recently shown to have a role in cancer initiation and progression. Jain showed that ZNF367 is overexpressed in adrenocortical carcinoma, malignant pheochromocytoma, paraganglioma, and thyroid cancer as compared to normal tissue and benign tumors. H. Zeng’s study indicated that ZNF367-induced transcriptional activation of KIF15 accelerates the progression of breast cancer (12), and X. Wu found that ZNF 367 promotes metastasis by inhibiting the Hippo pathway in breast cancer (13). However, some studies have shown that ZNF367 plays a tumor-suppressive role in tumors. The research results show that ZNF367 inhibits cellular proliferation, invasion, migration, and adhesion to extracellular proteins *in vitro* and *in vivo* (14). Dewaele illustrated that EWSR1-CREB3L3 gene fusion is related to a mesenteric sclerosing epithelioid fibrosarcoma (15). H. Liu et al. not only identified the close relationship between HEYL and tumor microenvironment phenotype but also emphasized the crucial importance of HEYL, which could be identified as a candidate biomarker to evaluate prognostic risk and therapeutic effect in gastric cancer (16).

The results of our study indicate that FOXD1, TIGD1, SNAI1, HSF4 were closely related to the prognosis of colon cancer. P. Quintero-Ronderos showed that molecular, structural, and functional aspects of FOXD1 are presented in light of physiological and pathogenic conditions, including its role in human disease etiology, such as cancer and recurrent pregnancy loss (17). L. Yin illustrated that TIGD1, a gene of unknown function, involves cell-cycle progression and correlates with poor prognosis in human cancer (18). D. Li’s analysis revealed that SNAI1 mRNA expression may potentially be a negative prognostic factor in breast cancer (19). J. Qi’s research indicated SNAI1 promotes the development of hepatocellular carcinoma (HCC) through the enhancement of proliferation and inhibition of apoptosis (20). Y. Yang found that high HSF4 expression is an independent indicator of poor overall survival and recurrence-free survival in patients with primary colorectal cancer (21).

The results of our study indicate that CENPA, ETS2, MYBL2, FOXM1, ETV4 were closely related to the prognosis of HCC. Y. Zhang’s study revealed that CENPA mRNA were upregulated in HCC patients with alpha-fetoprotein (AFP) elevation, advanced TNM stage, larger tumor size, advanced AJCC stage, advanced pathology grade, and vascular invasion. A Cox regression model including CENPA could predict OS in HCC patients effectively. CENPA might be an oncogenic factor in the development of HCC patients (22). According to literature reports, ETS2 can not only promote the development of tumors but also inhibit the development of tumors. M. Kabbout’s findings pointed to a tumor suppressor role for ETS2 in human non-small-cell lung cancer (NSCLC) pathogenesis through inhibition of the MET proto-oncogene (23). Y. L. Liao’s data indicated that ETS2 plays a key role in controlling the expression of miR-196b, and miR-196b may mediate the tumor suppressor effects of ETS2 (24). But X. Liu opined that ETS2 functions as an oncogene and plays a key role in the progression of hypopharyngeal cancer (25). L. Y. found that ETS2 knockdown inhibits tumorigenesis in esophageal squamous cell carcinoma *in vivo* and *in vitro* (26). G. W. Zhang revealed that downregulation of ETS2 inhibits the invasion and metastasis of renal cell carcinoma cells by inducing EMT *via* the PI3K/Akt signaling pathway (27). J. Zhu illustrated that MicroRNA-146b overexpression promotes human bladder cancer invasion *via* enhancing ETS2-mediated mmp2 mRNA transcription (28). J. Munera revealed that ETS2 could regulate colonic stem cells and sensitivity to tumorigenesis (29). Z. Guan found that high MYBL2 expression and transcription regulatory activity is associated with poor overall survival in patients with HCC (30). J. Dai’s research indicated that overexpression of FOXM1 was to the disadvantage of the prognosis for majority of solid tumor and therefore can be used as an evaluation index of prognosis (31). E. Kim revealed that capicua inhibited the progression of HCC by controlling the ETV4-MMP1 axis (32). Q. X.’s study indicated that PBK overexpression promotes metastasis of HCC *via* activating ETV4-uPAR signaling pathway (33).

The results of our study indicate that FOXQ1 and ZNF589 were closely related to the prognosis of pancreatic cancer. Forkhead Box Q1 (FOXQ1) is a member of the Forkhead Box protein family, which is a transcription factor with the function of regulating cell differentiation. In recent years, more and more studies have shown that FOXQ1 is significantly correlated with the pathogenesis of tumors. In many studies, upregulation of FOXQ1 expression has been reported in breast, colorectal, pancreatic, bladder, and ovarian cancers, as well as glioma, among other tumor types (34). ZNF589 is rarely reported in tumors. Oleksiewicz revealed that TRIM28 employs KRAB-ZNFs to evoke epigenetic silencing of its target differentiation genes *via* H3K9me3 and DNA methylation (35).

In summary, we analyzed the prognostic TFs in digestive system carcinoma based on the TCGA database, and then established a prognostic prediction model, which was verified successfully using the GEO database. TFs may affect the clinical prognosis of patients through different regulatory mechanisms. The above-mentioned literature suggests that proper regulation of TFs can benefit patients with digestive system carcinoma. Prognostic TFs in risk score models may provide new ideas for exploring therapeutic targets for digestive system carcinoma. Identification of appropriate TFs as therapeutic targets with further verification to ensure the clinical efficacy and safety on digestive system carcinoma patients would be a promising strategy in future studies. However, the molecular mechanism of TFs and the role of related signal transduction pathways in digestive system carcinoma remain unclear, which requires further research and exploration (6).

## Data Availability Statement

Publicly available datasets were analyzed in this study. This data can be found here: TCGA database (https://portal.gdc.cancer.gov/), GEO database (http://ncbi.nlm.nih.gov/geo/).

## Author Contributions

Study concept and design: GF, JF, ZD, YZ, JL. Acquisition, analysis, or interpretation of data: All authors. Statistical analysis: GF. Establishment of the model: GF. Drafting of the manuscript: GF. Critical revision of the manuscript for important intellectual content: All authors. Administrative, technical, or material support: YZ, JL. All authors contributed to the article and approved the submitted version.

## Funding

This work was supported by Jieping Wu Medical Foundation (LDWJPMF-102-17007), Medical Innovation Project of Fujian Province (2018-CX-49), and Specific Foundation of Development and Reform Commission in Fujian Province (31010308).

## Conflict of Interest

The authors declare that the research was conducted in the absence of any commercial or financial relationships that could be construed as a potential conflict of interest.

## Publisher’s Note

All claims expressed in this article are solely those of the authors and do not necessarily represent those of their affiliated organizations, or those of the publisher, the editors and the reviewers. Any product that may be evaluated in this article, or claim that may be made by its manufacturer, is not guaranteed or endorsed by the publisher.

## Supplementary Material

Supplementary Figure 1 Differential TFs between cancer tissue and paracancer tissue. **(A, B)** Heatmap **(A)** and Volcano plot **(B)** of the differential TFs in cancer tissue and paracancer tissue of TCGA database.

Supplementary Figure 2 The construction of TFs signature and the evaluation of its independent prognostic value. **(A, B)** Forest plot of the univariate Cox regression analysis with TFs in liver cancer and pancreatic cancer. **(C, D)** Forest plots of the univariate Cox regression analysis with clinical features and risk score in TCGA cancer cohorts.

Supplementary Figure 3 Heatmap, characteristics of the risk score, and Kaplan-Meier analysis of the OS-related TFs in training cohorts (TCGA database). **(A, B)** Heatmap of the gene-expression profiles of OS-related TFs in training cohorts: **(A)** liver cancer, **(B)** pancreatic cancer. **(C, D)** The distributions of the risk score, survival time, and status of patients in training cohorts: **(C)** liver cancer, **(D)** pancreatic cancer. **(E, F)** Kaplan-Meier curves of OS-related TFs in training cohorts: **(E)** liver cancer, **(F)** pancreatic cancer.

Supplementary Figure 4 Heatmap, characteristics of the risk score, and Kaplan-Meier analysis of the OS-related TFs in validation cohorts (GEO database). **(A, B)** Heatmap of the gene-expression profiles of OS-related TFs in validation cohorts: **(A)** liver cancer, **(B)** pancreatic cancer. **(C, D)** The distributions of the risk score, survival time, and status of patients in validation cohorts: **(C)** liver cancer, **(D)** pancreatic cancer. **(E, F)** Kaplan-Meier curves of OS-related TFs in validation cohorts: **(E)** liver cancer, **(F)** pancreatic cancer.

Supplementary Figure 5 Enrichment plot of the OS-related TFs between the high-risk and low-risk groups using GSEA. **(A, B)** The enriched gene sets in KEGG collection in liver cancer sample **(A)** and pancreatic cancer sample **(B)** with high risk score and low risk score.

Supplementary Figure 6 Prognostic capacity evaluation and nomogram analysis of panel of OS-TFs of digestive system carcinoma patients. **(A, B)** Nomogram predicting the OS in digestive system carcinoma patients containing the risk score: **(A)** liver cancer, **(B)** pancreatic cancer. **(C)** ROC curves and AUC for 1-, 3, and 5-year survival of the nomogram in liver cancer. **(D)** ROC curves and AUC for 1-, 2, and 3-year survival of the nomogram in pancreatic cancer. **(E, F)** Calibration curve of 3-year survival in the nomogram and ideal model: **(E)** liver cancer, **(F)** pancreatic cancer.

Supplementary Figure 7 The results of qRT-PCR in OS-related TFs. (A–E) CENPA, MYBL2, FOXM1, and ETV4 were highly expressed and ETS2 were lowly expressed in liver cancer tissues. (F, G) FOXQ1 and ZNF589 were highly expressed in pancreatic cancer tissues.

Supplementary Figure 8 Functional enrichment analysis of the target genes of OS-related TFs. **(A, B)** GO enrichment analysis results, showing only the first 20 terms in liver cancer **(A)** and pancreatic cancer **(B)**. **(C, D)** KEGG pathway enrichment analysis results, showing only the first 20 pathways in liver cancer **(C)** and pancreatic cancer **(D)**. Gene Ratio refers to the ratio of the number of genes enriched in the term/pathway to the total number of genes in the term/pathway.

## Abbreviations

TFs, transcription factors; TCGA, the cancer genome atlas; GEO, gene expression omnibus; FPKM, fragments per kilobase million; DEG, differential expression gene; FDR, false discovery rate; OS, overall survival; ROC, receiver operating characteristic; KM, Kaplan–Meier; GSEA, gene set enrichment analysis; RT-PCR, reverse transcription polymerase chain reaction; GO, gene ontology; KEGG, Kyoto encyclopedia of genes and genomes; HR, hazard ratio; 95% CI, 95% confidence interval; AUC, area under the curve; HCC, hepatocellular carcinoma.
