# Supplementary figures and images for: Prognostic and Predictive Value of Transcription Factors Panel for Digestive System Carcinoma
Source: Front Oncol. 2021 Oct 21;11:670129. doi: 10.3389/fonc.2021.670129 (PMC8566925; doi:10.3389/fonc.2021.670129)

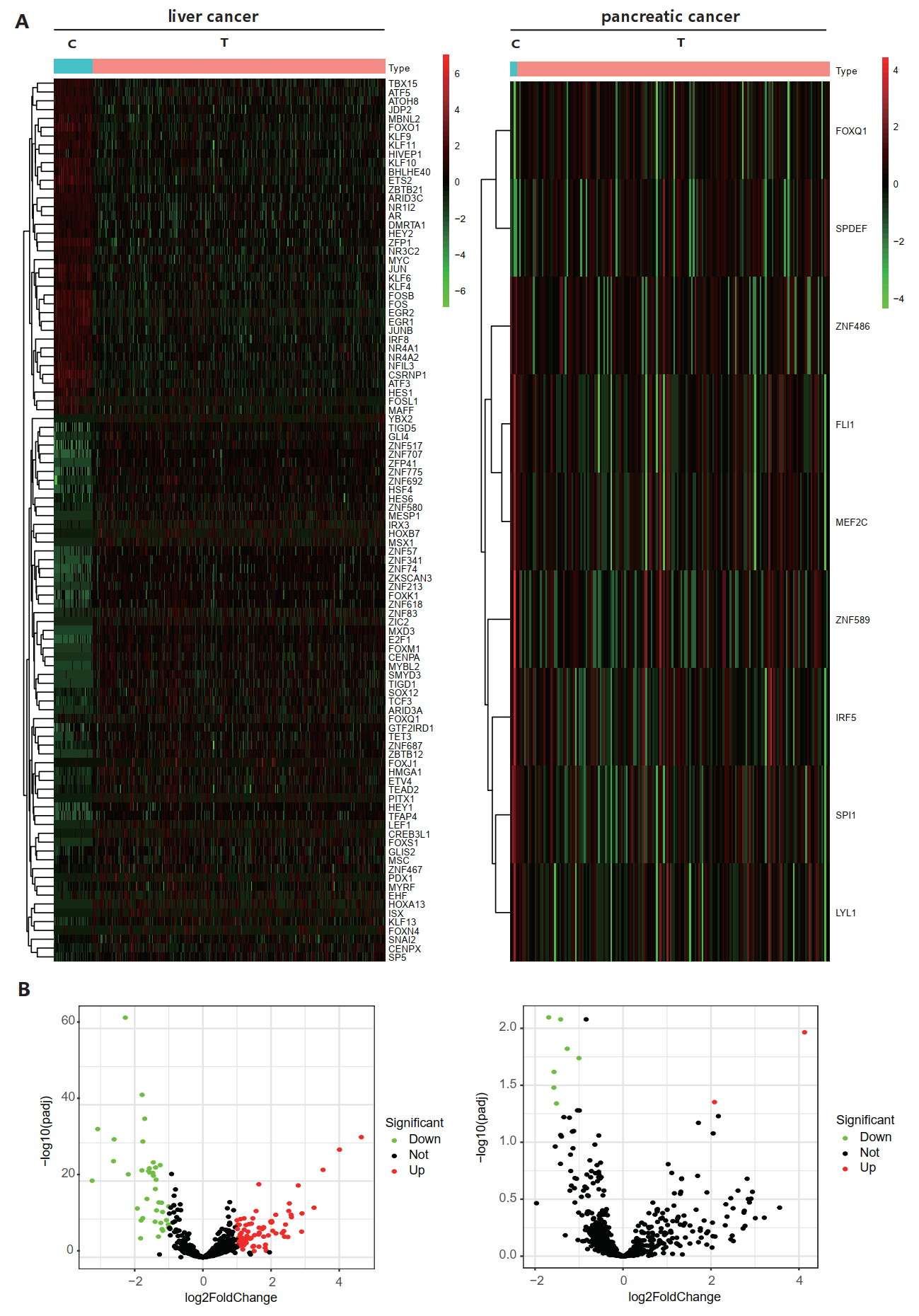

Supplement: Supplementary Figure 1 — Differential TFs between cancer tissue and paracancer tissue. (A, B) Heatmap (A) and Volcano plot (B) of the differential TFs in cancer tissue and paracancer tissue of TCGA database. [file Image_1.jpeg]

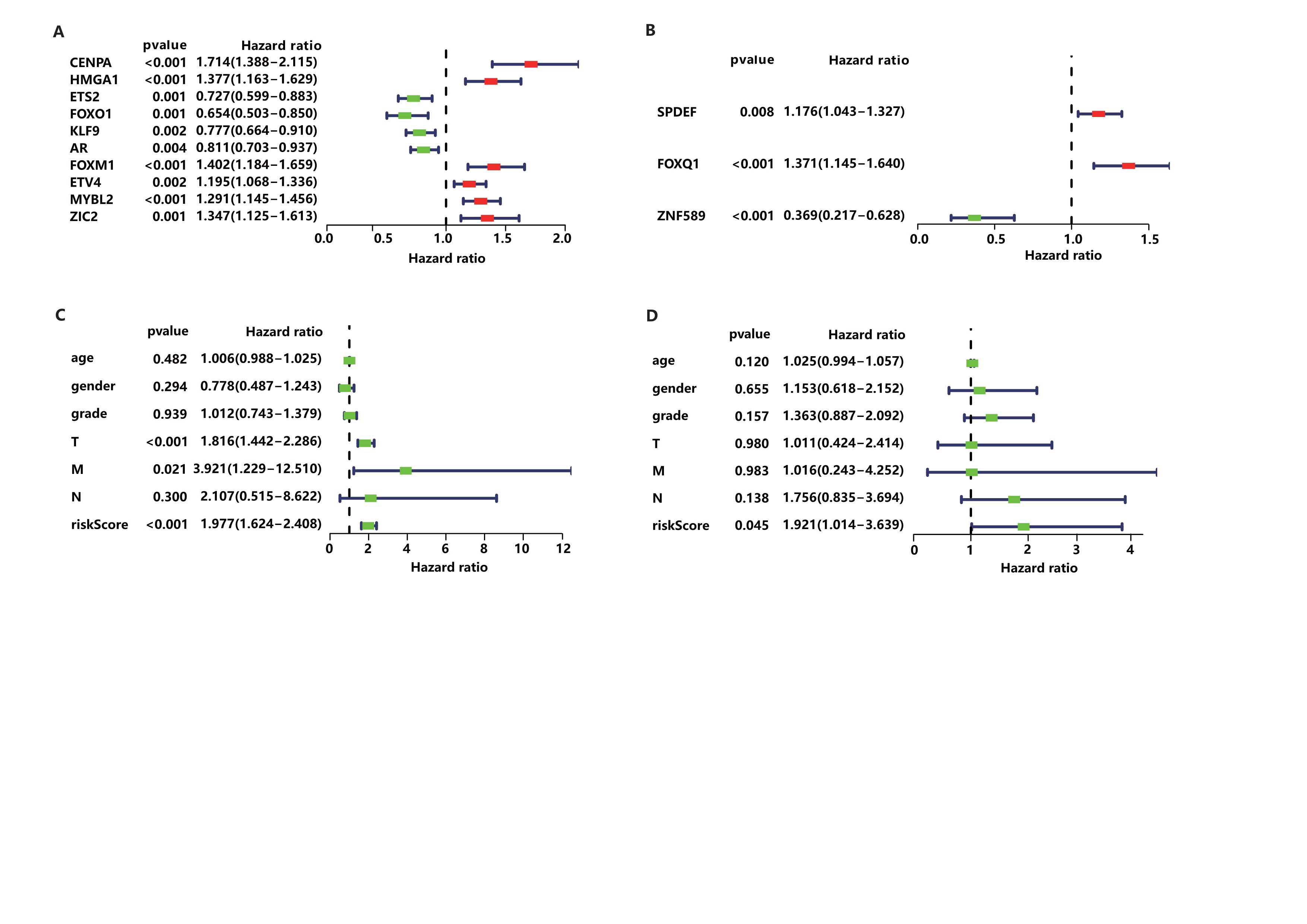

Supplement: Supplementary Figure 2 — The construction of TFs signature and the evaluation of its independent prognostic value. (A, B) Forest plot of the univariate Cox regression analysis with TFs in liver cancer and pancreatic cancer. (C, D) Forest plots of the univariate Cox regression analysis with clinical features and risk score in TCGA cancer cohorts. [file Image_2.jpeg]

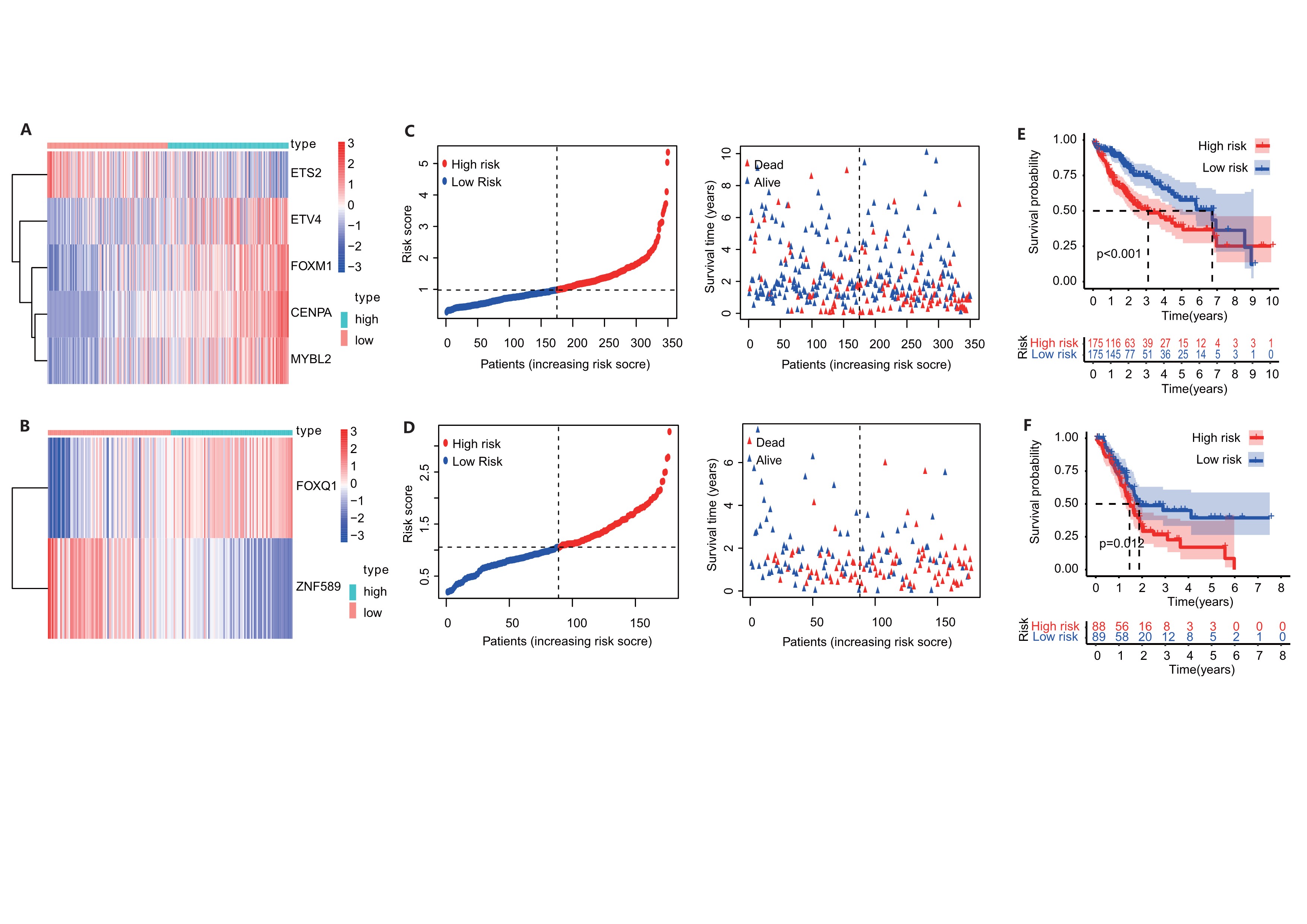

Supplement: Supplementary Figure 3 — Heatmap, characteristics of the risk score, and Kaplan-Meier analysis of the OS-related TFs in training cohorts (TCGA database). (A, B) Heatmap of the gene-expression profiles of OS-related TFs in training cohorts: (A) liver cancer, (B) pancreatic cancer. (C, D) The distributions of the risk score, survival time, and status of patients in training cohorts: (C) liver cancer, (D) pancreatic cancer. (E, F) Kaplan-Meier curves of OS-related TFs in training cohorts: (E) liver cancer, (F) pancreatic cancer. [file Image_3.jpeg]

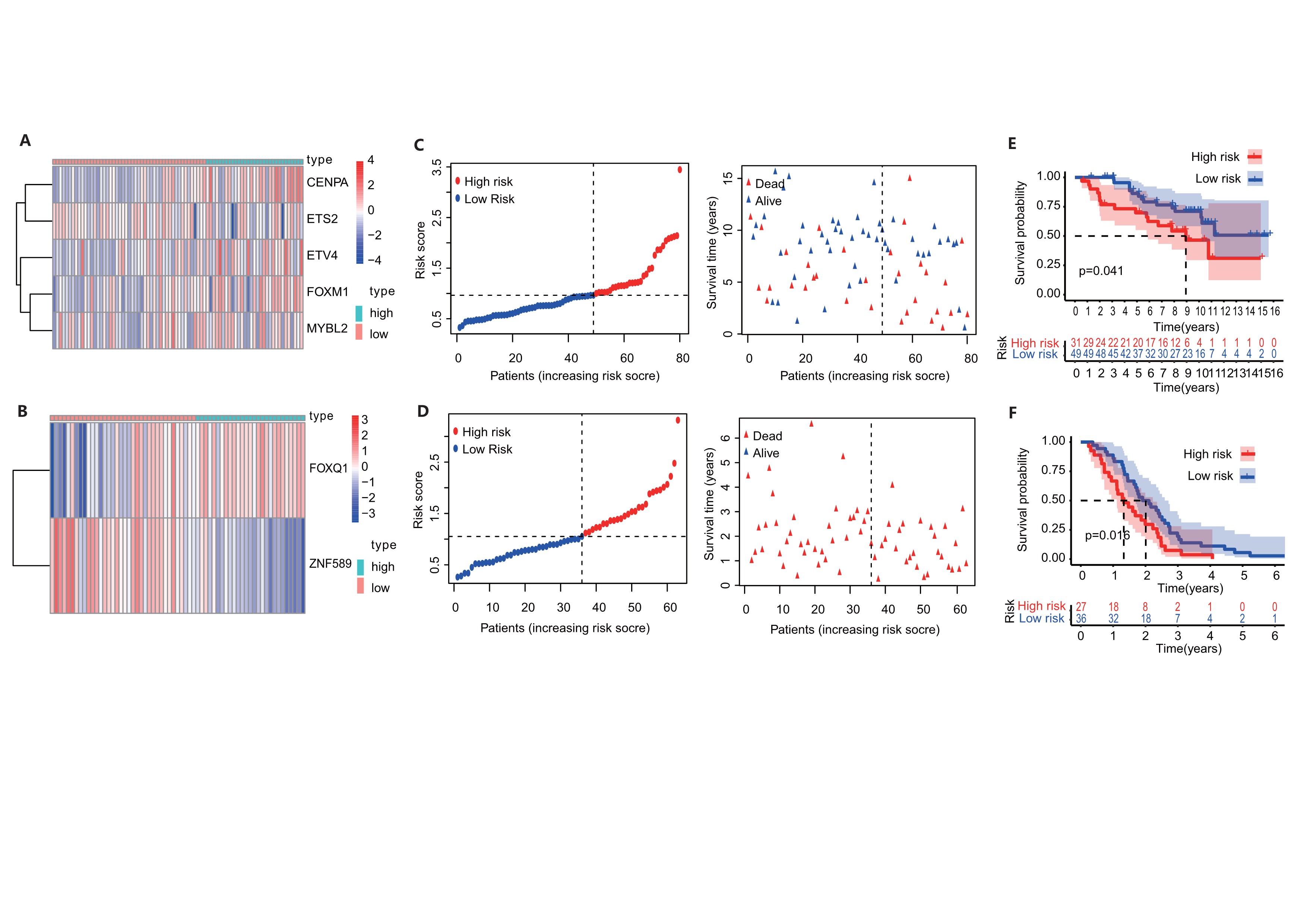

Supplement: Supplementary Figure 4 — Heatmap, characteristics of the risk score, and Kaplan-Meier analysis of the OS-related TFs in validation cohorts (GEO database). (A, B) Heatmap of the gene-expression profiles of OS-related TFs in validation cohorts: (A) liver cancer, (B) pancreatic cancer. (C, D) The distributions of the risk score, survival time, and status of patients in validation cohorts: (C) liver cancer, (D) pancreatic cancer. (E, F) Kaplan-Meier curves of OS-related TFs in validation cohorts: (E) liver cancer, (F) pancreatic cancer. [file Image_4.jpeg]

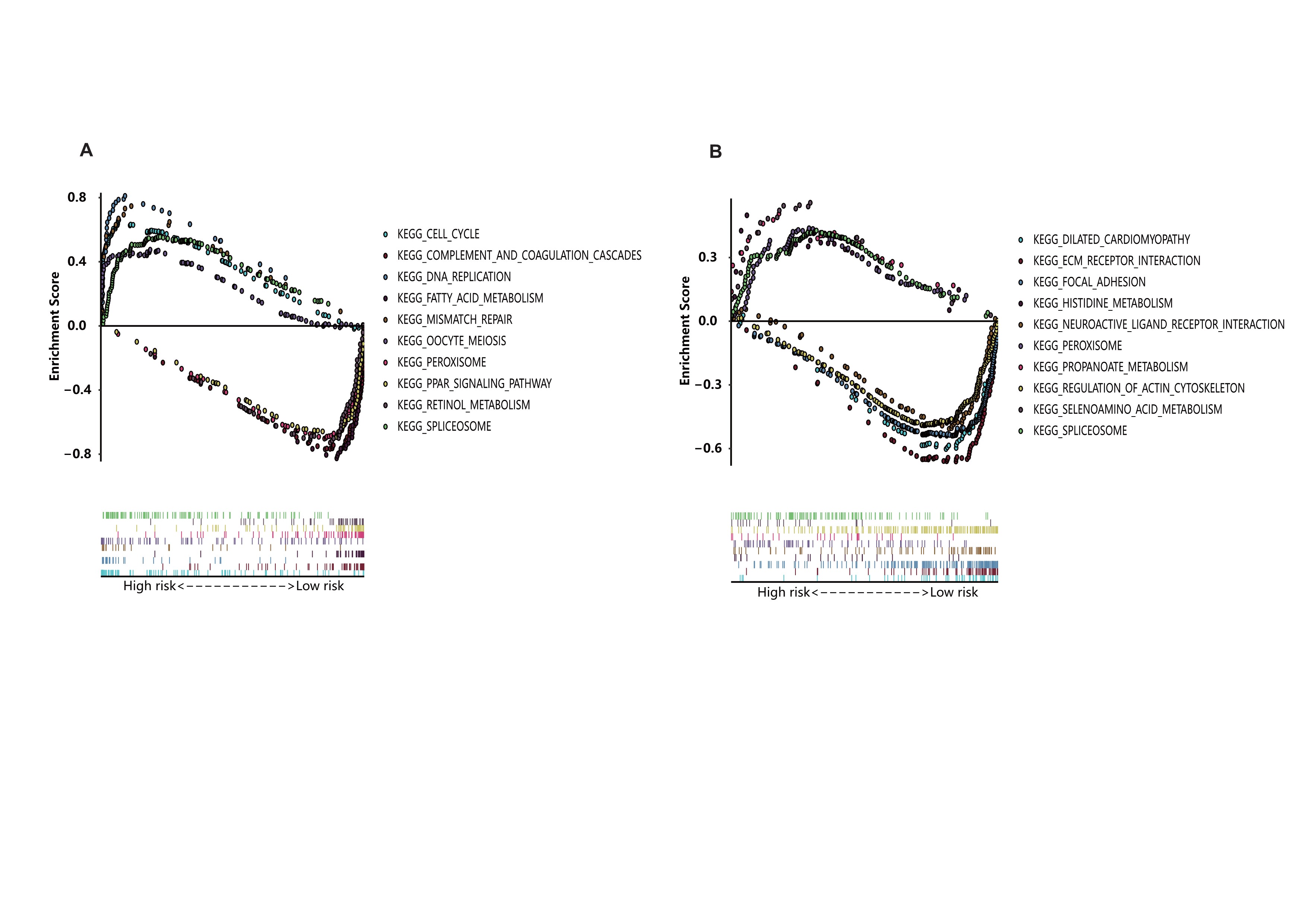

Supplement: Supplementary Figure 5 — Enrichment plot of the OS-related TFs between the high-risk and low-risk groups using GSEA. (A, B) The enriched gene sets in KEGG collection in liver cancer sample (A) and pancreatic cancer sample (B) with high risk score and low risk score. [file Image_5.jpeg]

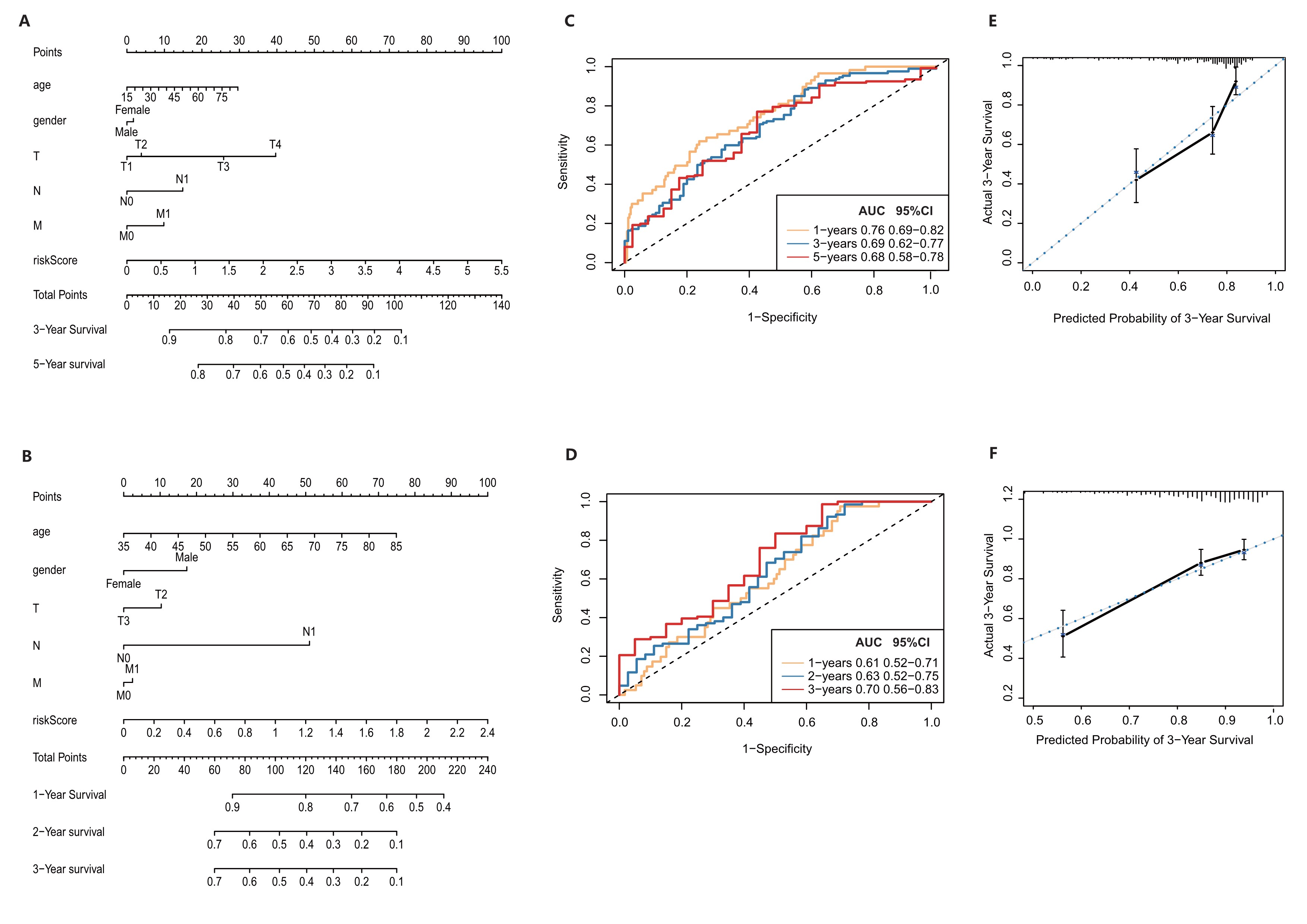

Supplement: Supplementary Figure 6 — Prognostic capacity evaluation and nomogram analysis of panel of OS-TFs of digestive system carcinoma patients. (A, B) Nomogram predicting the OS in digestive system carcinoma patients containing the risk score: (A) liver cancer, (B) pancreatic cancer. (C) ROC curves and AUC for 1-, 3, and 5-year survival of the nomogram in liver cancer. (D) ROC curves and AUC for 1-, 2, and 3-year survival of the nomogram in pancreatic cancer. (E, F) Calibration curve of 3-year survival in the nomogram and ideal model: (E) liver cancer, (F) pancreatic cancer. [file Image_6.jpeg]

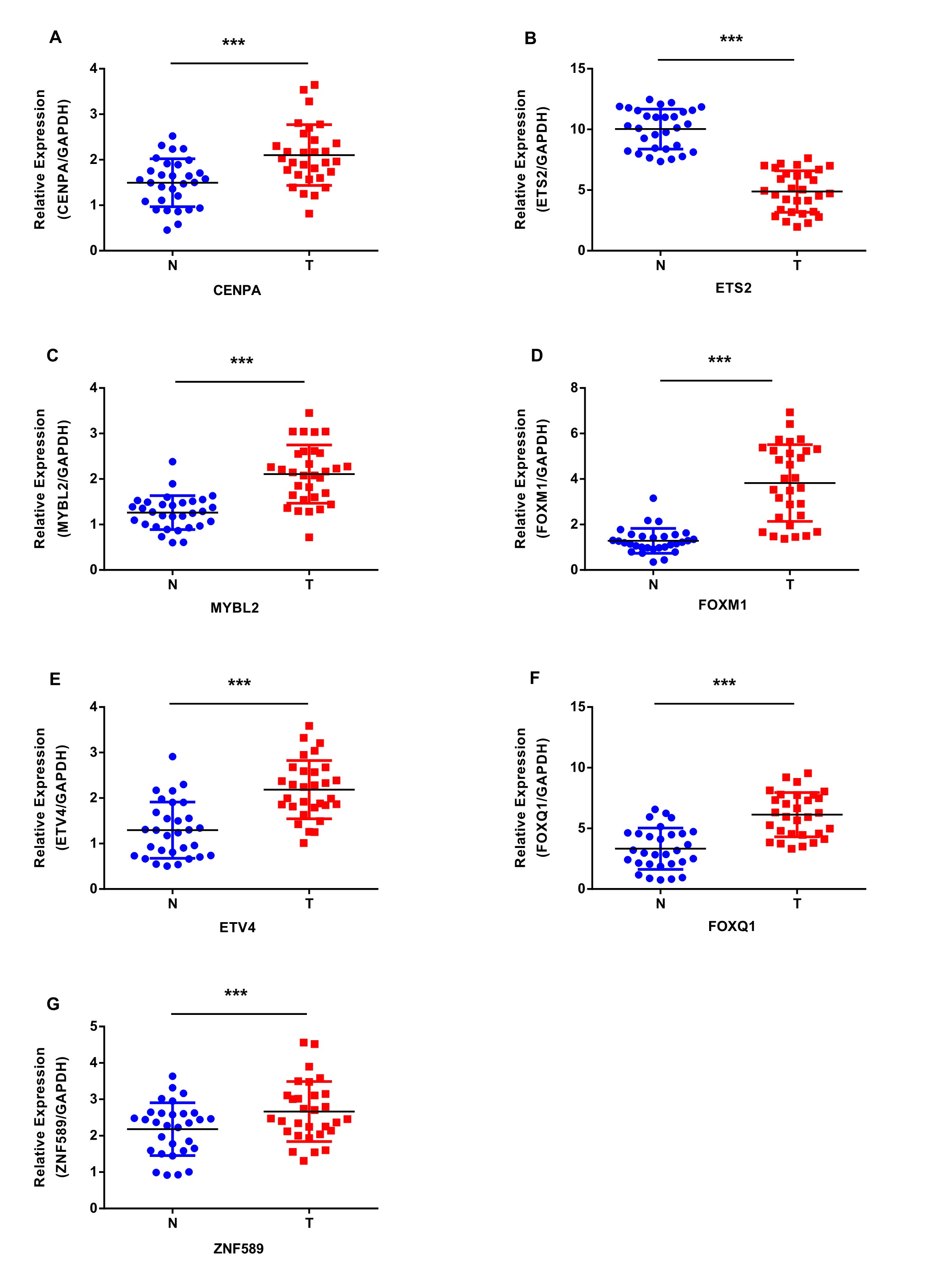

Supplement: Supplementary Figure 7 — The results of qRT-PCR in OS-related TFs. (A–E) CENPA, MYBL2, FOXM1, and ETV4 were highly expressed and ETS2 were lowly expressed in liver cancer tissues. (F, G) FOXQ1 and ZNF589 were highly expressed in pancreatic cancer tissues. [file Image_7.jpeg]

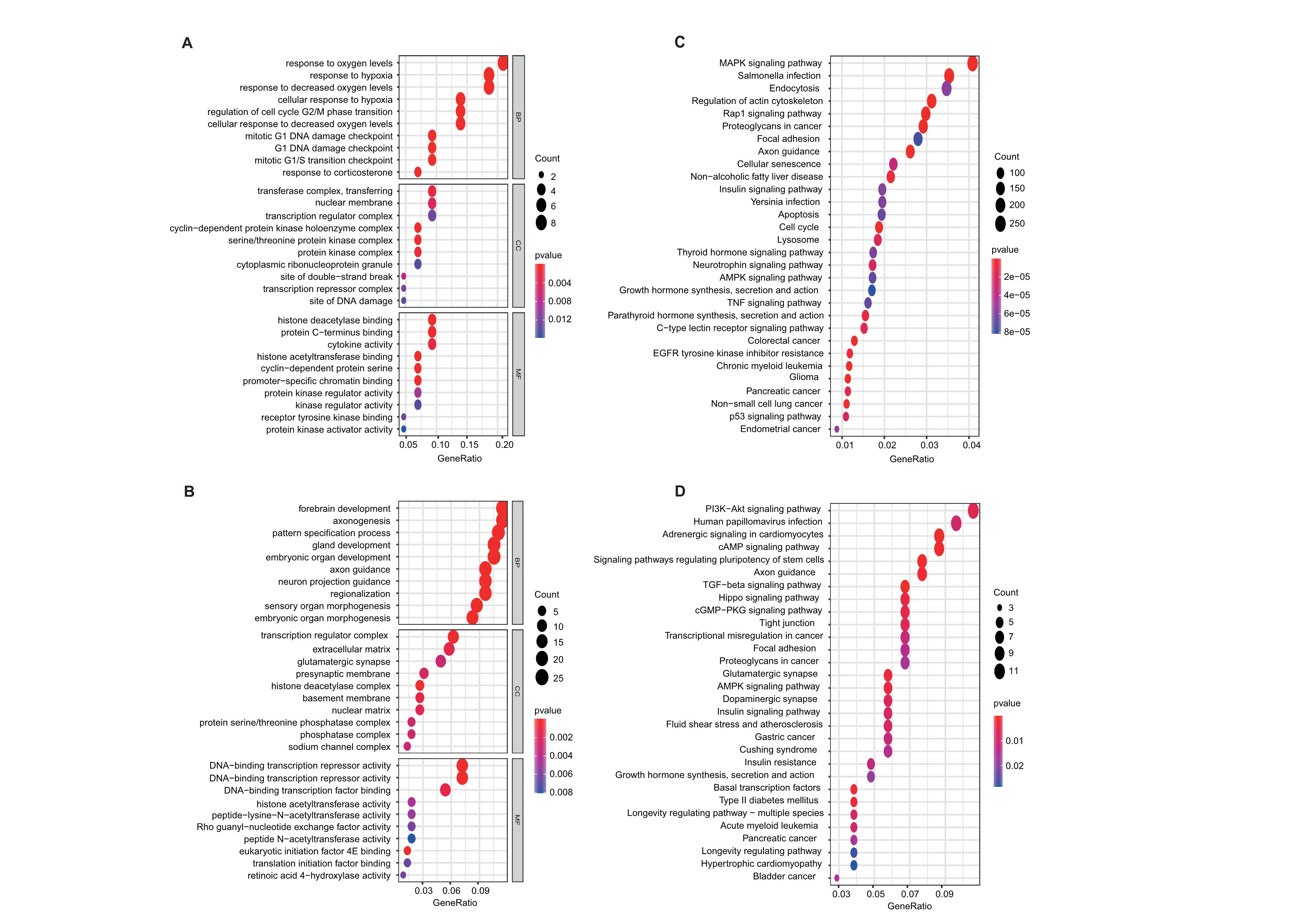

Supplement: Supplementary Figure 8 — Functional enrichment analysis of the target genes of OS-related TFs. (A, B) GO enrichment analysis results, showing only the first 20 terms in liver cancer (A) and pancreatic cancer (B). (C, D) KEGG pathway enrichment analysis results, showing only the first 20 pathways in liver cancer (C) and pancreatic cancer (D). Gene Ratio refers to the ratio of the number of genes enriched in the term/pathway to the total number of genes in the term/pathway. [file Image_8.jpeg]
